# Structural Studies of the Giant Mimivirus

**DOI:** 10.1371/journal.pbio.1000092

**Published:** 2009-04-28

**Authors:** Chuan Xiao, Yurii G Kuznetsov, Siyang Sun, Susan L Hafenstein, Victor A Kostyuchenko, Paul R Chipman, Marie Suzan-Monti, Didier Raoult, Alexander McPherson, Michael G Rossmann

**Affiliations:** 1 Department of Biological Sciences, Purdue University, West Lafayette, Indiana, United States of America; 2 Department of Molecular Biology and Biochemistry, University of California, Irvine, California, United States of America; 3 Unité des Rickettsies, Faculté de Médecine (CNRS) UMR 6020, IFR 48, Marseille, France; University of Wisconsin-Madison, United States of America

## Abstract

Mimivirus is the largest known virus whose genome and physical size are comparable to some small bacteria, blurring the boundary between a virus and a cell. Structural studies of Mimivirus have been difficult because of its size and long surface fibers. Here we report the use of enzymatic digestions to remove the surface fibers of Mimivirus in order to expose the surface of the viral capsid. Cryo-electron microscopy (cryoEM) and atomic force microscopy were able to show that the 20 icosahedral faces of Mimivirus capsids have hexagonal arrays of depressions. Each depression is surrounded by six trimeric capsomers that are similar in structure to those in many other large, icosahedral double-stranded DNA viruses. Whereas in most viruses these capsomers are hexagonally close-packed with the same orientation in each face, in Mimivirus there are vacancies at the systematic depressions with neighboring capsomers differing in orientation by 60°. The previously observed starfish-shaped feature is well-resolved and found to be on each virus particle and is associated with a special pentameric vertex. The arms of the starfish fit into the gaps between the five faces surrounding the unique vertex, acting as a seal. Furthermore, the enveloped nucleocapsid is accurately positioned and oriented within the capsid with a concave surface facing the unique vertex. Thus, the starfish-shaped feature and the organization of the nucleocapsid might regulate the delivery of the genome to the host. The structure of Mimivirus, as well as the various fiber components observed in the virus, suggests that the Mimivirus genome includes genes derived from both eukaryotic and prokaryotic organisms. The three-dimensional cryoEM reconstruction reported here is of a virus with a volume that is one order of magnitude larger than any previously reported molecular assembly studied at a resolution of equal to or better than 65 Å.

## Author Summary

Mimiviruses are larger than any other known virus, yet despite their size, the capsid has been shown to be a regular icosahedron. Using cryo-electron microscopy and atomic force microscopy, we show that the icosahedral symmetry is only approximate, in part because one of the 5-fold vertices has a unique “starfish-shaped” feature and because a better three-dimensional reconstruction was obtained by assuming only 5-fold symmetry. Contrary to expectations, the arrangement of the capsomers on the Mimivirus surface is not as that in many other large icosahedral dsDNA viruses. Instead, the faces of Mimivirus have systematic vacant sites that are surrounded by six capsomers with alternative orientations which differ by about 60°.

## Abstract 3

The structure of Mimivirus was examined with cryo-electron microscopy and atomic force microscopy. The quasi-icosahedral virus has a unique vertex decorated by a starfish-like feature. The capsomers form hexagonal arrays on each face.

## Introduction

Mimivirus, *Acanthamoeba polyphaga Mimivirus*, is the largest known virus [1–3] and a putative human pneumonia agent [4]. It has an icosahedral shape with a 0.75-μm diameter [3] and a ∼1.2-Mbp genome that contains most of the genes found in small bacteria [5]. The external morphology of Mimivirus had initially led to its false identification as a bacterium [1,4]. Initial cryo-electron microscopy (cryoEM) studies [3] had shown that Mimivirus has a diameter of about 5,000 Å, with multiple layers of proteins and lipid membranes that surround a nucleocapsid. In addition, there is a dense layer of 1,250-Å-long fibers that cover the viral surface, making the total diameter of the particles about 7,500 Å. The outermost layer of the capsid is about 70 Å thick and corresponds to the major capsid protein (MCP). There is an irregularly shaped nucleocapsid, which itself is enveloped by a 70-Å-thick layer, and is separated from the capsid by a distance that varies from 300 to 500 Å. Thus, the large size of Mimivirus, its gene content, and its functional complexity as described here and elsewhere [2–6] stretch the definition of a virus [7].

**Figure 1:**
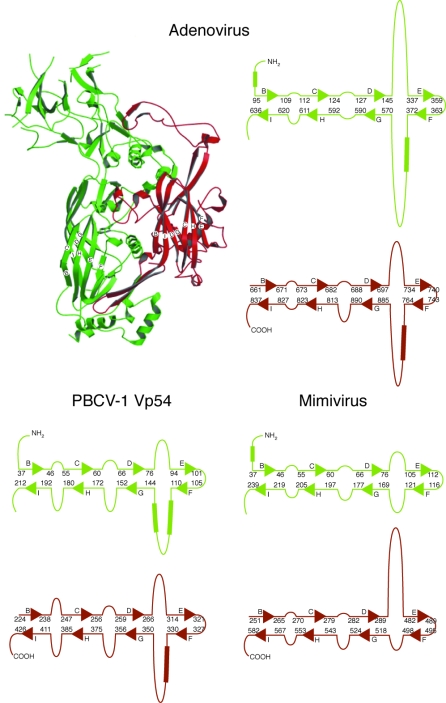
Comparison of Virus MCPs The N-terminal and C-terminal jelly-roll domains are colored green and red, respectively. Top left is a ribbon diagram of the adenovirus capsid protein. Diagrammatic representation of the arrangement of the β strands (arrows) within each jelly-roll are given for adenovirus, PBCV1, and Mimivirus at the top right, bottom left, and bottom right, respectively. The β strands within each domain are labelled A to H. This gives rise to the two opposing BIDG and CHEF β sheets in each jelly-roll as indicated in the ribbon diagram. Occasional α helices are represented as bars.

**Figure 2:**
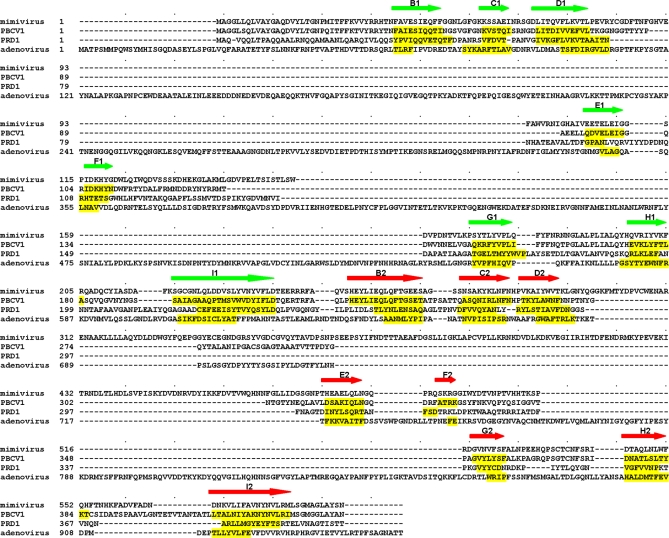
Sequence Alignments of Virus MCPs Sequence alignments of Mimivirus (gene product L425 [5]), PBCV1, PRD1, and adenovirus MCPs based on structural superpositions in all cases except Mimivirus. The β strands in the first and second jelly-roll motifs are labelled in green and red, respectively, with the corresponding sequences highlighted in yellow.

The capsomer structures of some large double-stranded DNA (dsDNA) viruses—including adenovirus [8], *Paramecium bursaria Chlorella* virus 1 (PBCV1) [9,10], the bacteriophage PRD1 [11], *Sulfolobus* turreted icosahedral virus [12], and the marine bacteriophage PM2 [13]—have been determined by x-ray crystallography and shown to be similar. Although these viruses infect a wide variety of hosts covering the prokaryotic, eukaryotic, and archaeal domains of life, the similarity of their MCP structures suggest that they have, in part, evolved from a common precursor [9,12,14]. Each monomer in the trimeric capsomers consists of two successive “jelly-roll” folds, producing a pseudo-hexameric structure with a thickness of ∼75 Å and a diameter varying between 74 Å in PBCV1 [9,10] and about 85 Å in adenovirus [8]. One or other of the two jelly-roll motifs within the monomer often has a large insertion in the DE and FG loops (the β strands along the polypeptide of each jelly-roll are named A to H) (Figures 1 and 2), creating a “tower” on top of each of the three monomers within a capsomer. These towers give capsomers a triangular appearance on the surface while maintaining a pseudo-hexagonal shape below the towers, appropriate for packing into hexagonal arrays [8,15,16]. The Mimivirus MCP is homologous to the MCP of PBCV1 with 31% amino acid identity (Figure 2). Therefore, it is highly likely that the structure of Mimivirus capsomers are similar to the aforementioned capsomers in large dsDNA icosahedral viruses [9,12,14]. However, in Mimivirus, there are about 190 additional amino acids inserted into the DE loop of the second jelly-roll motif that are similar to the large tower insertions in adenovirus (Figures 1 and 2).

The forest of long fibers on the Mimivirus surface increases the ice thickness, creating difficulties for cryoEM [3]. The random scattering of the electrons by the additional ice thickness and by the disordered fibers reduced the signal-to-noise ratio. Here we report that we were able to partially overcome this problem by digesting the fibers with lysozyme and proteases. Both atomic force microscopy (AFM) and cryoEM were then used to analyze the structure of untreated as well as defibered Mimiviruses. The viral capsid surface was found to have a hexagonal array of depressions separated by about 140 Å. These were interpreted as systematic vacancies within a hexagonal array of double jelly-roll capsomers, accounting for the absence of one-third of all capsomers. Furthermore, the previously recognized starfish-like feature [17] was well-resolved and associated with a unique pentameric vertex on mature particles below the forest of surface fibers. We also show that the Mimivirus nucleocapsid has a defined shape surrounded by an envelope, which is separated from the viral capsid by a space whose size and dimensions are conserved in all particles.

## Results

### The 5-Fold Symmetry of Mimivirus

**Figure 3:**
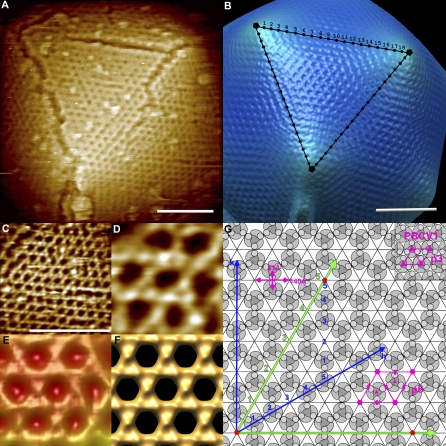
Capsomer Arrangement of Mimivirus (A) AFM image of defibered virus showing one face of the virion with irregular cracks along the edges of the face. (B) Shaded surface rendering of the cryoEM reconstruction computed from untreated Mimivirus, orientated similarly as in (A). The surface fibers are not visible because of their disorder. Depressions at the edges of a face are marked with dots. The dots along one edge are labelled from 1 to 18. Three 5-fold vertices are labelled with black pentagons. (C) An AFM image of defibered particle at high magnification shows a honeycomb array of the depressions. (D) Enlargement of a portion of (C). (E) CryoEM reconstruction of fibered particles on the same scale as (D). (F) Mimivirus surface simulated using the homologous PBCV1 capsomer structure and the observed spatial arrangement of the capsomers in (D) and (E). (G) Diagram showing the *p*6 plane group lattice of capsomers as found in (D) and (E). Each monomer of the MCP consists of two consecutive jelly-rolls indicated by a kidney-shaped outline with a circle at one end representing the inserted “tower” loop. Three monomers constitute one capsomer. The depressions along one edge are shown in green. Three 5-fold vertices at the corners of a face are shown with red pentagons. As an example the 5-fold vertices are placed 5 depressions apart. This would correspond to *h* = 5 and *k* = 5 in terms of the Caspar-Klug *p*3 plane group hexagonal array of capsomers (see blue axes). For Mimivirus, there are 19 depressions along an edge. One unit cell of the *p*6 (Mimivirus) and *p*3 (PBCV1) are outlined in red. The distance between depressions (140 Å) and between capsomers (81 Å) is shown in red. The scale bars in (A), (B), and (C) represent 1,000 Å.

**Figure 4:**
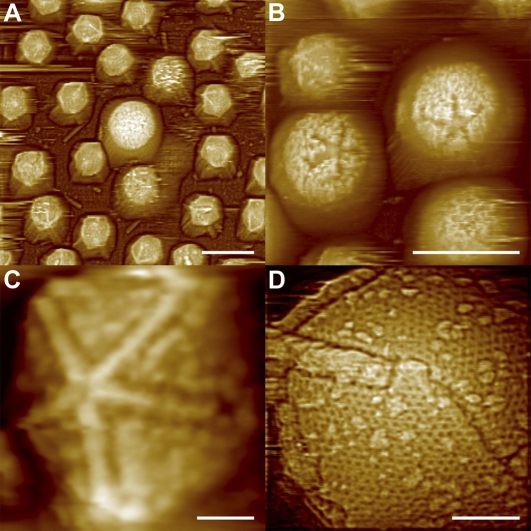
AFM Images of Starfish-shaped Features on Defibered Mimiviruses (A) Defibered Mimivirus at low magnification showing that the majority of the particles have starfish-shaped features. Presumably, the few particles that do not show the starfish-shaped feature are orientated with their special vertex towards the substrate. The fibers on a particle in the middle of the micrograph were not fully digested, as is evident by its larger diameter. (B) Partially digested Mimivirus at median magnification. One particle on the upper-left corner is fully digested and shows a starfish-shaped feature. The other three particles have retained their fibers and have a larger diameter with star-shaped crevices between the fibers, which demonstrates that the starfish-shaped feature has no attached fibers. (C) A high-magnification image of a starfish-shaped feature on a defibered virus. (D) A defibered particle treated with proteinase K showing the arm of a starfish-shaped feature wedged into the 2-fold edge creating a 500 Å separation between arrays of capsomers. The scale bars in (A) and (B) represent 1 μm, and those in (C) and (D) are 1,000 Å.

A large number of cryoEM particle images were collected to improve the previously computed [3], icosahedrally averaged, three-dimensional reconstructions of Mimivirus. However, increasing the number of images beyond about 30,000 failed to show the anticipated hexagonal arrays of capsomers as found in PBCV1 and other related dsDNA viruses [12,18,19]. Hence, AFM was used in an endeavor to obtain better-resolved structural information. AFM images of defibered Mimivirus showed hexagonal arrays of depressions covering the surface of the virus (Figure 3A) and a starfish-like structure associated with one of the vertices on many of the particles (Figure 4), as had also been observed on some previous EM micrographs [17]. The presence of a structural feature on only one of the 12 vertices demonstrated a significant departure from icosahedral symmetry. Therefore, further reconstructions were based on only 5-fold, rather than icosahedral symmetry (see Materials and Methods), using about 700 lysozyme- and protease-treated defibered virus particles. This reconstruction clearly showed a unique pentameric vertex with a starfish-like attachment, but failed to visualize the hexagonal arrays of depressions seen on the AFM images. Because about 31,000 images of the mature fibered particles had been collected, a further reconstruction was calculated—using these particles and assuming only 5-fold symmetry—which was initialized with the newly reconstructed model from the defibered particles. The resultant 65 Å resolution cryoEM map of Mimivirus showed that surface depressions, separated by 140 Å, were arranged in hexagonal arrays (Figure 3B), which was consistent with the AFM observations. Each equilateral triangular face of the virion consisted of 19 rows of depressions parallel to each edge, with each row containing one less depression than the previous row.

### The Mimivirus Capsid Organization

The Mimivirus genome [5] contains four genes, including *L425* and *R441*, that are homologous to the double jelly-roll PBCV1 Vp54, and to the MCPs of other large dsDNA viruses [14]. Although a homology model of the *R441* gene product was built by Benson et al. [14], the actual MCP of Mimivirus was found to be the gene product of *L425* [5]. The limited resolution of the cryoEM reconstruction barely resolves individual capsomers, but the array of large depressions suggests that these are missing capsomers (vacancies) in the hexagonal arrays of PBCV1-like capsomers. There is one 190-amino-acid-long insertion in the DE loop of the second jelly-roll along the polypeptide of the Mimivirus MCP (Figures 1 and 2), which is located on the external edge of each of the three monomers in a capsomer. The systematic vacancies in Mimivirus could arise as a consequence of steric conflict between these insertions in three neighboring capsomers and would be relieved by creating the systematic vacancies.

Each of the depressions on the Mimivirus surface is surrounded by six barely resolved triangular shapes (Figure 3C–3E), which are similar in appearance to the triangular external surface of capsomers in other viruses with double jelly-roll MCPs [8,15,16]. The orientations of neighboring trimeric capsomers surrounding each depression differ by about 60°, thus generating a 6-fold symmetry axis in the center of each depression (Figure 3C–3F). However, the trimeric shapes are barely resolved from each other so that each of the three “towers” that form the triangular shape at the top of a capsomer merges with the towers of the neighbouring capsomers (Figure 3G). A simulation using the known PBCV1 capsomer structure [9], assembled into hexagonal arrays as found for Mimivirus, demonstrated that the proposed arrangement mimics the observed pattern of depressions with poorly resolved surrounding trimeric capsomers at the resolution attained for the Mimivirus reconstruction (Figure 3F). Given that the distance between depressions is 140 Å, the center-to-center distance between adjacent triangular capsomers will be 81 Å (Figure 3G). This is in the range expected for trimeric capsomers assembled from double jelly-roll monomers [10].

## Discussion

### The *T* Number of Mimivirus

To our knowledge, the arrangement of protein subunits in an icosahedral capsid was first discussed by Crick and Watson [20]. Their concepts were extended by Caspar and Klug, who suggested that arrays of hexagonal capsomers could be interspersed with pentameric capsomers at the icosahedral 5-fold vertices, resulting in only quasi-equivalent environments for monomers at the 5-fold vertex compared with those in hexagonal arrays [21]. The organization of pseudo-hexameric capsomers in large dsDNA icosahedral viruses, for which the triangulation number (*T*) expresses the number of jelly-rolls rather than monomers in the icosahedral asymmetric unit, is, therefore, a further extension of the concept of quasi-symmetry.

If all the depressions were filled by capsomers, and as there are 19 depressions between neighboring pentameric vertices, the coordinates of the nearest vertex would be *h* = 19 ± 1 and *k* = 19 ± 1, where *h* and *k* are the number of capsomers along the hexagonal axes of the array (Figure 3G). The uncertainty arises because it is not clear whether there is a depression or a capsomer on each pentameric vertex. Thus, the triangulation number, given by *T* = *h* ^2^ + *hk* + *k* ^2^ [21], would be 3 × (19 ± 1)^2^ or have one of nine possible values in the range of 972 ≤ *T* ≤ 1200. The previously predicted value of around 1,180 jelly rolls [3] was based on an estimate for the center-to-center distance between capsomers being 75 Å. The above observations show that this distance is 81 Å, which would have given *T* = 1,012, which is still within the range of the above determination. However, this statement further extends the definition of *T*, because it not only considers the depressions being filled by capsomers, but also tacitly assumes that all the capsomers are similarly oriented. The *p*6 plane group arrangement of capsomers in Mimivirus allows 3/2 times as much area per capsomer—compared with a completely filled hexagonal array of capsomers in a *p*3 plane group—as, for instance, in PBCV1 (Figure 3G). Thus, the actual number of jelly-rolls will be 2*T*/3, and the number of capsomers per icosahedral asymmetric unit will be *T*/9 or about 120 for Mimivirus.

The *p*6 plane group organization of the capsomers in Mimivirus is essentially the same as that of trimeric “packing units” observed by cryoEM for infectious bursal disease virus (IBDV) [22,23] which has a *T* = 13 (*h* = 1, *k* = 3) surface lattice. The structure of the IBDV major capsid protein has been determined [24] and shown to have three domains (B, S, and P), of which the S and P domains have jelly-roll folds. However, the domain organization within the IBDV trimeric capsomers is different to the pseudo-hexagonal capsomer structures found in PBCV1 and some other large dsDNA viruses. Thus, although the *p*6 organization of capsomers in Mimivirus resembles the capsid of a dsRNA virus, the amino acid sequence of the major capsid protein of Mimivirus has greatest similarity to other dsDNA viruses such as PBCV1.

### The Starfish-Shaped Feature and Its Possible Function in Genome Delivery

**Figure 5:**
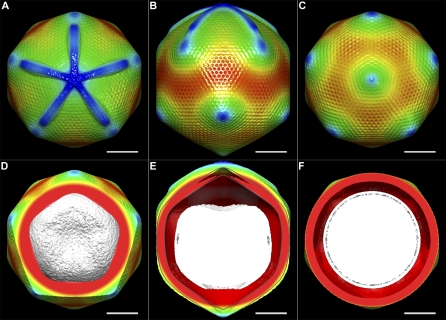
CryoEM Reconstruction of Mimivirus Applying Only 5-fold Symmetry Averaging (A–C) Surface-shaded rendering of cryoEM reconstruction of untreated Mimivirus. (A) Looking down the starfish-shaped feature associated vertex, (B) looking from one side, and (C) looking from the opposite side of the “starfish”-associated vertex. (D) The “starfish”-associated vertex was removed to show the internal nucleocapsid with its concave surface facing the special vertex. (E) Central slice of the reconstruction looking from the side of the particle showing the concave face of the nucleocapsid and the low density space beneath the “starfish”-associated vertex. A perfectly icosahedral particle is outlined in gray to show the extension of the unique vertex. (F) Central slice of the reconstruction looking along the 5-fold axis from the starfish-shaped feature showing the enveloped nucleocapsid surrounded by a lower density space. The coloring is based on radial distance from the center of the virus. Gray is from 0 to 1,800 Å, red from 1,800 to 2,100 Å, and rainbow coloring from red to blue between 2,100 and 2,500 Å. The scale bars in all panels represent 1,000 Å.

**Figure 6:**
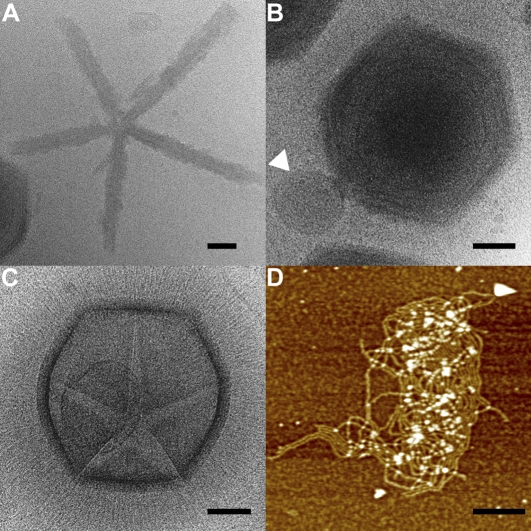
Function of the Starfish-Shaped Feature The features shown here have been observed reproducibly and frequently in numerous images. (A) CryoEM image of a starfish-shaped feature that has become detached from the virus. (B) CryoEM image of a defibered Mimivirus that has lost its starfish-shaped feature. A “puff” (white arrow), surrounded by a membrane-like envelope [17,25] is observed at the special vertex. (C) CryoEM image of a Mimivirus showing a gap (less density represented by lighter gray shading) between five fibered faces surrounding the open unique vertex. The actual starfish-shaped feature and probably the viral nucleocapsid are missing. (D) AFM image of DNA ejected from Mimivirus. No other molecules appear to be protecting the ejected DNA (see Materials and Methods). Scale bar represents 1,000 Å in all panels.

CryoEM studies of Mimivirus recognized that some particles had a special vertex [3]. More recently, transmission electron microscopy (TEM) of sectioned Mimivirus-infected amoeba found a starfish-shaped feature associated with one vertex on many Mimiviruses [17]. Starfish-shaped density features were also observed with cryoEM on some fiberless immature Mimivirus particles that occurred in purified samples [17]. Here we show, using AFM, that a starfish-shaped feature can be seen on many defibered Mimivirus particles (Figure 4). Furthermore, the 5-fold–averaged cryoEM reconstruction of Mimivirus was initiated with a simplified model (see Materials and Methods) that did not have a starfish-shaped feature. However, the resulting reconstruction (Figure 5) showed a starfish-shaped feature similar to what was observed with AFM (Figure 4C) and confirmed the existence of the starfish-shaped feature on each virus. The 5-fold–averaged cryoEM results showed that the arms of the starfish have a thickness of about 400 Å, a width of about 500 Å, and extend about 2,000 Å almost all the way towards the neighbouring 5-fold vertices. The exceptional clarity of the starfish-shaped feature in cryoEM reconstructed map (Figure 5) demonstrated that it must exist on almost every fibered particle. Both AFM and cryoEM showed that the arms of the starfish are inserted and open a gap between the neighbouring faces that are associated with the special vertex (Figure 4D). The five faces associated with the special vertex are inclined by about 5° to what would be expected if the virus were completely icosahedral, accounting for the gap between faces (Figure 5E). The arms of the starfish-shaped feature do not show the hexagonal arrays of depressions (Figure 4D), suggesting that the starfish-like feature is not assembled from the MCP. Evidence for the starfish-shaped feature being a separate entity was also found in cryoEM images of defibered Mimivirus samples in which there were objects that had five arms of appropriate size radiating from a common center (Figure 6A).

CryoEM images of thin sectioned samples [17] and AFM images of mature Mimivirus (Figure 4B) showed that there are star-shaped crevices between the long surface fibers, implying that the starfish-shaped feature is not covered by fibers. It had been suggested that the “starfish”-associated vertices might be the portal for DNA release based on its location further from the associated virus factory [17,25]. Thus, if the long cross-linked fibers of Mimiviruses [3] were to cover the complete viral surface, they would be an obstacle for genome delivery into a host. However, the star-shaped crevice between the fibers could provide an exit portal for the genome.

Scanning electron microscopy [17], traditional TEM of thin sections [25], and cryoEM studies (Figure 6B) show that defibered particles missing the starfish-shaped feature are associated with membrane-like “puffs” at their special vertices. Furthermore, cryoEM showed that particles that had lost their genome (Figure 6C) had also lost the starfish-shaped feature. In addition, AFM showed that the ejected DNA is unprotected by any surrounding proteins (Figure 6D). Thus, the starfish-shaped feature might be acting as a seal to hold together the five faces associated with the special vertex. Therefore, the first step of genome delivery would be the release of the starfish-shape feature, allowing the DNA to exit through the special vertex. Special vertices for genome delivery have also been observed in some other large dsDNA viruses [26,27], in tailed bacteriophages [28–31], and in herpes virus [32]. The presence of a special vertex in tailed bacteriophages or in herpes virus whose MCPs have a HK97-like fold [33] or in viruses that have a double jelly-roll fold in their capsids, suggests convergent evolution to a common solution for genome delivery.

### The Surface Fibers

**Figure 7:**
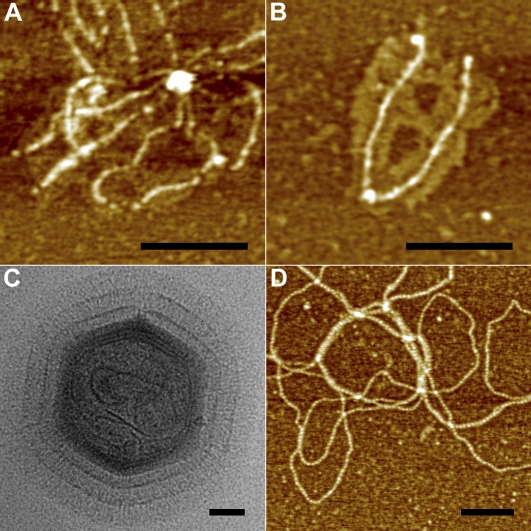
Mimivirus Fibers (A) AFM image of several surface fibers attached to a common central feature. Each of the fibers has a globular density at its free end. (B) AFM image of two detached surface fibers of Mimivirus. Murky material surrounding the fibers might be peptidoglycans [5] consistent with the uptake of Gram stain [1,4]. (C) CryoEM image of a Mimivirus that has been digested with lysozyme and then partially digested with bromelain. There are two successive rings of densities on the fibers. (D) AFM image of internal fibers of Mimivirus. The repeat distance between subunit is ∼70 Å, different to the repeating unit of DNA. Furthermore, the organization of the fibers is different to that of viral DNA as seen in Figure 6D. Scale bar represents 1,000 Å in all panels.

AFM images show that a number of external fibers of Mimivirus are frequently attached to a single central feature at one end with their free end being associated with a globular terminus (Figure 7A and 7B). However, there is no indication where the fibers attach to the capsid on the viral surface. The surface fibers are resistant to proteases unless first treated with lysozyme, suggesting that the fibers are protected by peptidoglycan (as previous suggested [5]), which is consistent with Mimivirus being Gram-positive [1,4]. CryoEM images of Mimivirus that had been partially treated with bromelain show successive rings of density on the fibers separated by 200–500 Å, representing different structural segments along their lengths (Figure 7C). AFM images show murky material surrounding the fibers (Figure 7B) that might be peptidoglycan cross-linking neighboring fibers. Fibers with peptidoglycan components perhaps act as a decoy for attracting amoeba [34].

### Mimivirus Nucleocapsid

The central slice of the cryoEM reconstruction, perpendicular to the unique 5-fold axis, showed that the genome is surrounded by a membrane-like envelope (Figure 5F). A central slice, containing the unique 5-fold axis, showed that the nucleocapsid had a concave depression facing the “starfish”-associated vertex (Figure 5E), which suggests a specialized organization that might be required for host infection. The clarity of these features after five-fold averaging implies that the nucleocapsid has a defined shape and also a fixed position relative to the external capsid. Unlike many other viruses in which the genome is closely surrounded by the capsid, Mimivirus has a 300–500 Å gap between the enveloped genome and the outer capsid. Thus, there must be supports across the gap that accurately position the genome relative to the viral capsid and internal membrane, although apparently they are too few or lack symmetry to make them visible in the cryoEM reconstruction. Long internal fibers were observed by AFM after applying mechanical force to the virus that broke the outer capsid layers (see Materials and Methods). These internal fibers have a diameter of about 60 Å with repeat units at intervals of about 70 Å (Figure 7D). The nucleocapsid might be supported by these fibers but, at this time, there is no further evidence for this suggestion.

### Conclusions

The enveloped genome within the larger viral capsid, perhaps supported by fibers (Figure 7D), has some similarity to eukaryotic cells. In contrast, the external peptidoglycan component mimics bacterial cell walls (Figure 7A–7C). In addition, the existence of a unique vertex in Mimivirus, possibly for genome delivery [17,25], is reminiscent of tailed bacteriophages. These observations are consistent with other results [2,35], implying that Mimiviruses and some other large icosahedral dsDNA viruses have gathered genes from eukaryotic, prokaryotic, as well as archaeal origins.

The three-dimensional cryoEM reconstruction reported here, which was made possible in part by relaxing the icosahedral symmetry, is of a virus whose volume is an order of magnitude larger than has previously been reported. Thus, the detection of a unique vertex may have been missed in other structural studies in which strict icosahedral symmetry had been imposed [36].

## Materials and Methods

### Production of defibered Mimivirus.

The Mimivirus fibers were digested by sequential application of lysozyme and bromelain. The Mimivirus was pelleted by centrifuging at 1,000*g* for 30 min. Each volume of pelleted virus was incubated with four volumes of 10 mg/ml lysozyme in TES buffer (0.05 M N-[Tris(hydroxymethyl)methyl]-2-aminoethanesulfonic acid, pH 7.5, 0.01% NaN_3_) at room temperature for at least one day. The sample was washed twice with TES and digested with five volumes of 14 mg/ml bromelain from pineapple stem (Sigma) in TES buffer (0.035M TES, pH = 7.5, 0.3M KCl, 0.02M DTT) at room temperature for at least one day.

### CryoEM.

CryoEM data of untreated and defibered Mimivirus were collected as described previously [3]. Micrographs were scanned on a Nikon Coolscan 9000 with a final pixel size of 15.9 Å. The cryoEM reconstruction was performed assuming 5-fold symmetry using programs FREALIGN [37] and a modified version of XMIPP [38] (V.A. Kostyuchenko et al., unpublished data). The reconstruction was initiated with a model in which the density of the five faces around one pentameric vertex were pushed outwards along the associated icosahedral 5-fold axis by about 300 Å. Of a total of 1,378 boxed defibered Mimivirus particles, 691 were selected to produce a map of 120 Å resolution. The resolution was determined using a Fourier shell correlation threshold of 0.5. The map shows a clear starfish-shaped feature. This map was used as a starting model for reconstruction of untreated, fibered Mimivirus. Of a total of 53,640 boxed fibered Mimivirus particles, 30,919 were selected to achieve a 5-fold-averaged reconstruction with a resolution of 65 Å. The cryoEM map has been deposited with the EBI and has been given the accession number of EMDB 10623.

### AFM.

Mimivirus particles, both native and those treated with enzymes, were spread on freshly cleaved mica that was coated with poly-l-lysine and scanned under buffer. Capsids, which were pretreated with lysozyme and bromelain, were, in some experiments, further exposed to 1 mg/ml solutions of proteinase K and 1% SDS at 37 °C for 30 min to 2 h, washed with water, and then imaged. No fixation of any kind was used. Two methods were used to expel the DNA and other internal structures from the virus. In the first method, virus solution was dried on mica, rehydrated with a small amount of water, and then pressed between two surfaces of mica. In the second method, very concentrated virus solution was placed in small wells, crushed with a glass stick, diluted in water, and then deposited on mica. The Mimivirus DNA was recognized by comparing the AFM images with DNA extracted from PBCV1 [16], T4 phage, vaccinia viruses [39], plasmid DNA, and thymus DNA. All these images had the same tangled appearance and had the same height (thickness) above substrate. Furthermore, the material could not come from the host or some other source, because almost all the images show these fibers to be closely associated with isolated virions, not just spread out randomly on the substrate.

AFM analysis was carried out using a Nanoscope III multimode instrument (Veeco Instruments). Samples were scanned at 25 °C using oxide-sharpened silicon nitride tips in a 75-μl fluid cell containing buffer or in air. For scanning in air, silicon tips were used. The images were collected in tapping mode [40] with an oscillation frequency of 9.2 kHz in fluid and 300 kHz in air, with a scan frequency of 1 Hz. Procedures were fundamentally the same as described for previous investigations of viruses [16,41]. In the AFM images presented here, height above substrate is indicated by increasingly lighter color. Thus, points very close to the substrate are dark and those well above the substrate are white. Because lateral distances are distorted due to an AFM image being a convolution of the cantilever tip shape with the surface features scanned, quantitative measures of size were based either on heights above the substrate or on center-to-center distances on particle surfaces. The AFM instrument was calibrated to the small lateral distances by imaging the 111 face of a thaumatin protein crystal and using the known lattice spacings [42] as standard.

## Abbreviations

AFM: atomic force microscopy
cryoEM: cryo-electron microscopy
dsDNA: double-stranded DNA
IBDV: infectious bursal disease virus
MCP: major capsid protein
PBCV1: Paramecium bursaria *Chlorella* virus 1
TEM: transmission electron microscopy
